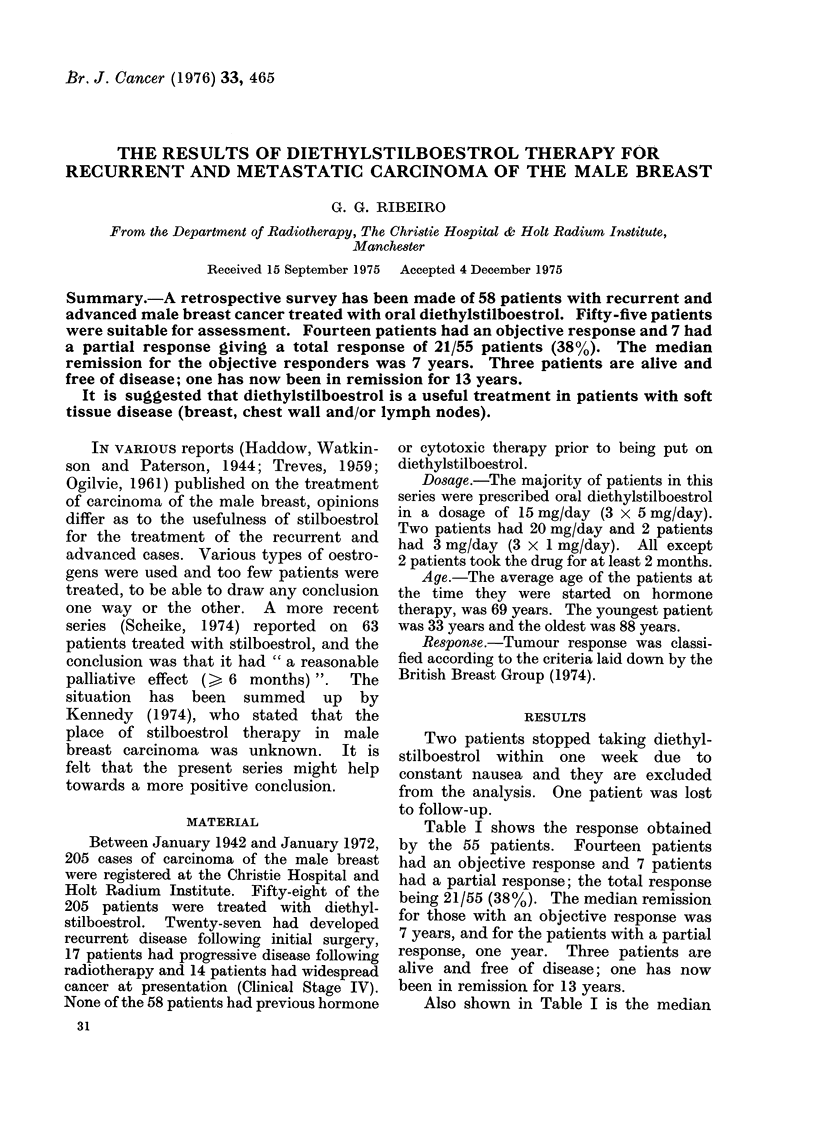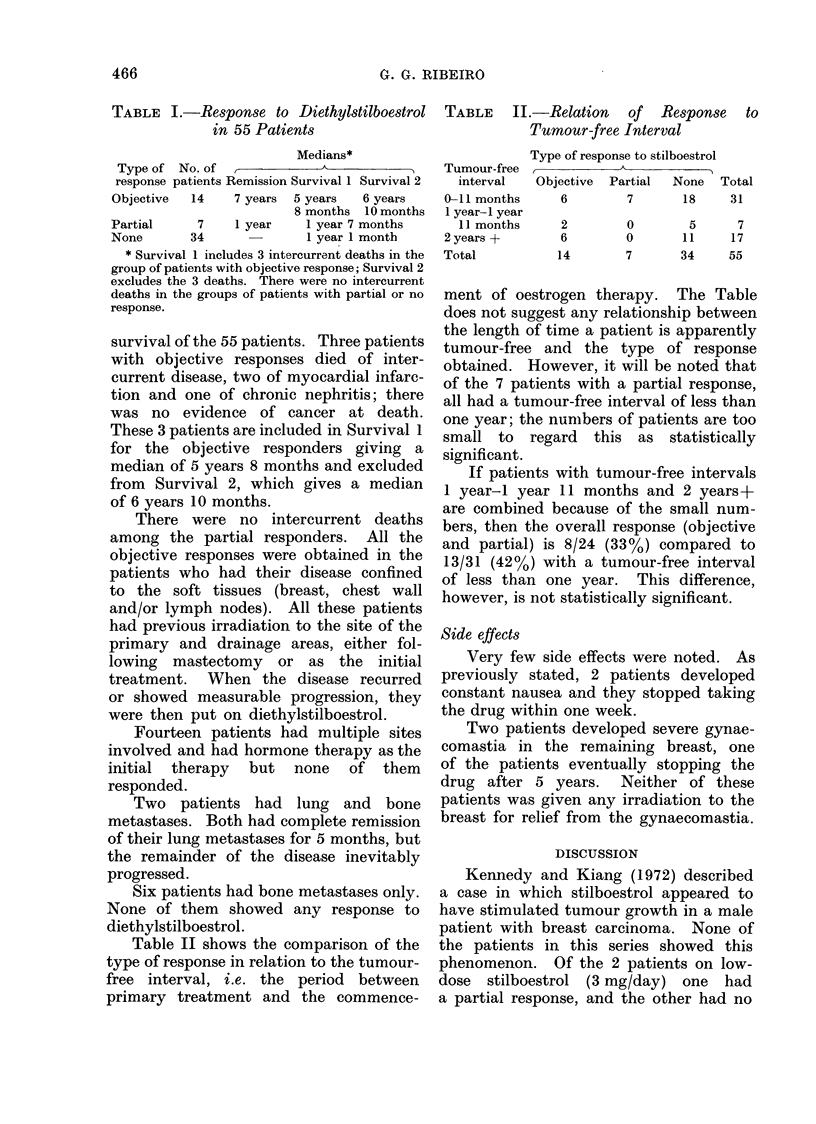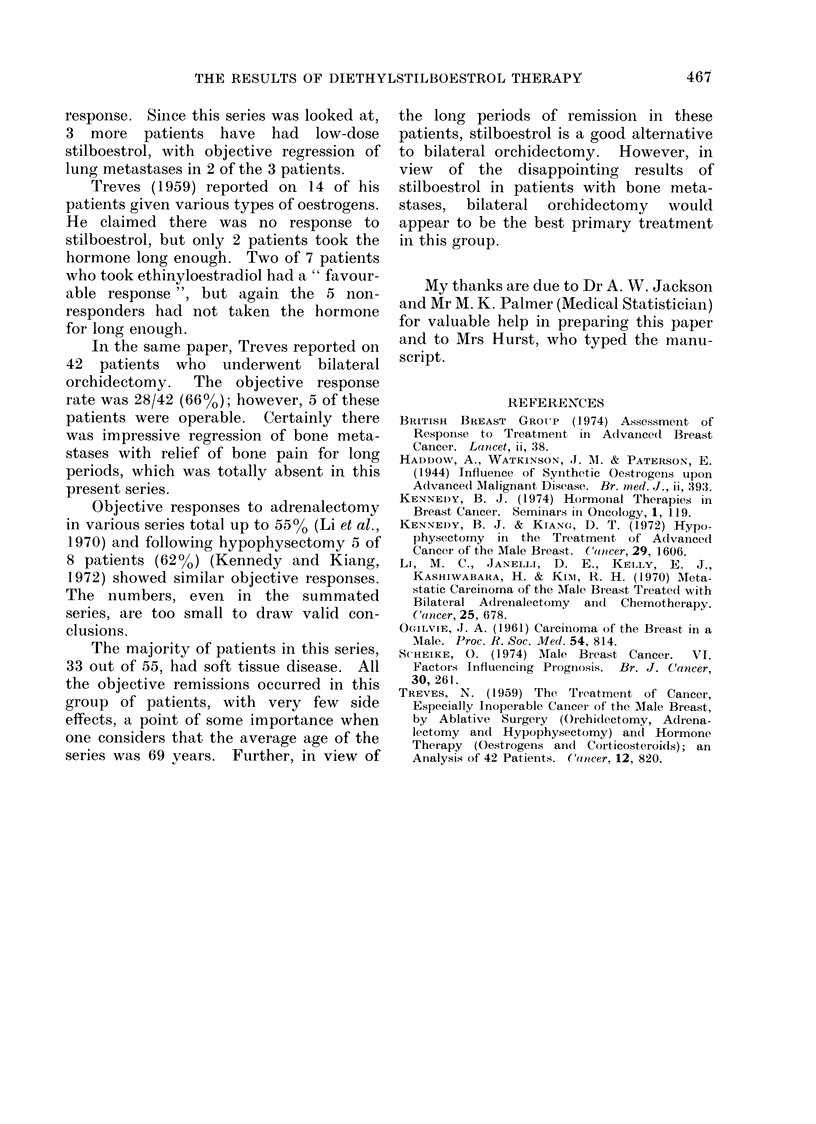# The results of diethylstilboestrol therapy for recurrent and metastatic carcinoma of the male breast.

**DOI:** 10.1038/bjc.1976.72

**Published:** 1976-04

**Authors:** G. G. Ribeiro

## Abstract

A retrospective survey has been made of 58 patients with recurrent and advanced male breast cancer treated with oral diethylstilboestrol. Fifty-five patients were suitable for assessment. Fourteen patients had an objective response and 7 had a partial response giving a total response of 21/55 patients (38%). The median remission for the objective responders was 7 years. Three patients are alive and free of disease; one has now been in remission for 13 years. It is suggested that diethylstilboestrol is a useful treatment in patients with soft tissue disease (breast, chest wall and/or lymph nodes).


Br. J. Cancer (1976) 33, 465

### THE RESULTS OF DIETHYLSTILBOESTROL THERAPY FOR RECURRENT AND METASTATIC CARCINOMA OF THE MALE BREAST

G. G. RIBEIRO

*From the Department of Radiotherapy, The Christie Hospital & Holt Radium Institute, Manchester*

Received 15 September 1975 Accepted 4 December 1975

**Summary.—A retrospective survey has been made of 58 patients with recurrent and advanced male breast cancer treated with oral diethylstilboestrol. Fifty-five patients were suitable for assessment. Fourteen patients had an objective response and 7 had a partial response giving a total response of 21/55 patients (38%). The median remission for the objective responders was 7 years. Three patients are alive and free of disease; one has now been in remission for 13 years.**

**It is suggested that diethylstilboestrol is a useful treatment in patients with soft tissue disease (breast, chest wall and/or lymph nodes).**

In various reports (Haddow, Watkinson and Paterson, 1944; Treves, 1959; Ogilvie, 1961) published on the treatment of carcinoma of the male breast, opinions differ as to the usefulness of stilboestrol for the treatment of the recurrent and advanced cases. Various types of oestrogens were used and too few patients were treated, to be able to draw any conclusion one way or the other. A more recent series (Scheike, 1974) reported on 63 patients treated with stilboestrol, and the conclusion was that it had " a reasonable palliative effect ($\geqslant$ 6 months) ". The situation has been summed up by Kennedy (1974), who stated that the place of stilboestrol therapy in male breast carcinoma was unknown. It is felt that the present series might help towards a more positive conclusion.

### MATERIAL

Between January 1942 and January 1972, 205 cases of carcinoma of the male breast were registered at the Christie Hospital and Holt Radium Institute. Fifty-eight of the 205 patients were treated with diethylstilboestrol. Twenty-seven had developed recurrent disease following initial surgery, 17 patients had progressive disease following radiotherapy and 14 patients had widespread cancer at presentation (Clinical Stage IV). None of the 58 patients had previous hormone or cytotoxic therapy prior to being put on diethylstilboestrol.

*Dosage.*—The majority of patients in this series were prescribed oral diethylstilboestrol in a dosage of 15 mg/day (3 × 5 mg/day). Two patients had 20 mg/day and 2 patients had 3 mg/day (3 × 1 mg/day). All except 2 patients took the drug for at least 2 months.

*Age.*—The average age of the patients at the time they were started on hormone therapy, was 69 years. The youngest patient was 33 years and the oldest was 88 years.

*Response.*—Tumour response was classified according to the criteria laid down by the British Breast Group (1974).

### RESULTS

Two patients stopped taking diethylstilboestrol within one week due to constant nausea and they are excluded from the analysis. One patient was lost to follow-up.

Table I shows the response obtained by the 55 patients. Fourteen patients had an objective response and 7 patients had a partial response; the total response being 21/55 (38%). The median remission for those with an objective response was 7 years, and for the patients with a partial response, one year. Three patients are alive and free of disease; one has now been in remission for 13 years.

Also shown in Table I is the median

TABLE I.—*Response to Diethylstilboestrol in 55 Patients*

| Type of response | No. of patients | Medians* Remission | Survival 1 | Survival 2 |
|---|---|---|---|---|
| Objective | 14 | 7 years | 5 years 8 months | 6 years 10 months |
| Partial | 7 | 1 year | 1 year 7 months | |
| None | 34 | — | 1 year 1 month | |

* Survival 1 includes 3 intercurrent deaths in the group of patients with objective response; Survival 2 excludes the 3 deaths. There were no intercurrent deaths in the groups of patients with partial or no response.

TABLE II.—*Relation of Response to Tumour-free Interval*

| Tumour-free interval | Type of response to stilboestrol Objective | Partial | None | Total |
|---|---|---|---|---|
| 0–11 months | 6 | 7 | 18 | 31 |
| 1 year–1 year 11 months | 2 | 0 | 5 | 7 |
| 2 years + | 6 | 0 | 11 | 17 |
| Total | 14 | 7 | 34 | 55 |

survival of the 55 patients. Three patients with objective responses died of intercurrent disease, two of myocardial infarction and one of chronic nephritis; there was no evidence of cancer at death. These 3 patients are included in Survival 1 for the objective responders giving a median of 5 years 8 months and excluded from Survival 2, which gives a median of 6 years 10 months.

There were no intercurrent deaths among the partial responders. All the objective responses were obtained in the patients who had their disease confined to the soft tissues (breast, chest wall and/or lymph nodes). All these patients had previous irradiation to the site of the primary and drainage areas, either following mastectomy or as the initial treatment. When the disease recurred or showed measurable progression, they were then put on diethylstilboestrol.

Fourteen patients had multiple sites involved and had hormone therapy as the initial therapy but none of them responded.

Two patients had lung and bone metastases. Both had complete remission of their lung metastases for 5 months, but the remainder of the disease inevitably progressed.

Six patients had bone metastases only. None of them showed any response to diethylstilboestrol.

Table II shows the comparison of the type of response in relation to the tumour-free interval, *i.e.* the period between primary treatment and the commencement of oestrogen therapy. The Table does not suggest any relationship between the length of time a patient is apparently tumour-free and the type of response obtained. However, it will be noted that of the 7 patients with a partial response, all had a tumour-free interval of less than one year; the numbers of patients are too small to regard this as statistically significant.

If patients with tumour-free intervals 1 year–1 year 11 months and 2 years+ are combined because of the small numbers, then the overall response (objective and partial) is 8/24 (33%) compared to 13/31 (42%) with a tumour-free interval of less than one year. This difference, however, is not statistically significant.

### *Side effects*

Very few side effects were noted. As previously stated, 2 patients developed constant nausea and they stopped taking the drug within one week.

Two patients developed severe gynaecomastia in the remaining breast, one of the patients eventually stopping the drug after 5 years. Neither of these patients was given any irradiation to the breast for relief from the gynaecomastia.

### DISCUSSION

Kennedy and Kiang (1972) described a case in which stilboestrol appeared to have stimulated tumour growth in a male patient with breast carcinoma. None of the patients in this series showed this phenomenon. Of the 2 patients on low-dose stilboestrol (3 mg/day) one had a partial response, and the other had no response. Since this series was looked at, 3 more patients have had low-dose stilboestrol, with objective regression of lung metastases in 2 of the 3 patients.

Treves (1959) reported on 14 of his patients given various types of oestrogens. He claimed there was no response to stilboestrol, but only 2 patients took the hormone long enough. Two of 7 patients who took ethinyloestradiol had a " favourable response ", but again the 5 non-responders had not taken the hormone for long enough.

In the same paper, Treves reported on 42 patients who underwent bilateral orchidectomy. The objective response rate was 28/42 (66%); however, 5 of these patients were operable. Certainly there was impressive regression of bone metastases with relief of bone pain for long periods, which was totally absent in this present series.

Objective responses to adrenalectomy in various series total up to 55% (Li *et al.*, 1970) and following hypophysectomy 5 of 8 patients (62%) (Kennedy and Kiang, 1972) showed similar objective responses. The numbers, even in the summated series, are too small to draw valid conclusions.

The majority of patients in this series, 33 out of 55, had soft tissue disease. All the objective remissions occurred in this group of patients, with very few side effects, a point of some importance when one considers that the average age of the series was 69 years. Further, in view of the long periods of remission in these patients, stilboestrol is a good alternative to bilateral orchidectomy. However, in view of the disappointing results of stilboestrol in patients with bone metastases, bilateral orchidectomy would appear to be the best primary treatment in this group.

My thanks are due to Dr A. W. Jackson and Mr M. K. Palmer (Medical Statistician) for valuable help in preparing this paper and to Mrs Hurst, who typed the manuscript.

### REFERENCES

British Breast Group (1974) Assessment of Response to Treatment in Advanced Breast Cancer. *Lancet*, ii, 38.

Haddow, A., Watkinson, J. M. & Paterson, E. (1944) Influence of Synthetic Oestrogens upon Advanced Malignant Disease. *Br. med. J.*, ii, 393.

Kennedy, B. J. (1974) Hormonal Therapies in Breast Cancer. Seminars in Oncology, **1**, 119.

Kennedy, B. J. & Kiang, D. T. (1972) Hypophysectomy in the Treatment of Advanced Cancer of the Male Breast. *Cancer*, **29**, 1606.

Li, M. C., Janelli, D. E., Kelly, E. J., Kashiwabara, H. & Kim, R. H. (1970) Metastatic Carcinoma of the Male Breast Treated with Bilateral Adrenalectomy and Chemotherapy. *Cancer*, **25**, 678.

Ogilvie, J. A. (1961) Carcinoma of the Breast in a Male. *Proc. R. Soc. Med.* **54**, 814.

Scheike, O. (1974) Male Breast Cancer. VI. Factors Influencing Prognosis. *Br. J. Cancer*, **30**, 261.

Treves, N. (1959) The Treatment of Cancer, Especially Inoperable Cancer of the Male Breast, by Ablative Surgery (Orchidectomy, Adrenalectomy and Hypophysectomy) and Hormone Therapy (Oestrogens and Corticosteroids); an Analysis of 42 Patients. *Cancer*, **12**, 820.